# Gender Disparities in Illegal Drug-Related Mortality: A National Ecological Study in Iran

**DOI:** 10.34172/jrhs.11593

**Published:** 2026-02-21

**Authors:** Mehran Rostami, Seyed Amirhosein Mahdavi, Mohammad Jalilian, Shahab Rezaeian

**Affiliations:** ^1^Deputy of Health, Kermanshah University of Medical Sciences, Kermanshah, Iran; ^2^Legal Medicine Research Center, Legal Medicine Organization, Tehran, Iran; ^3^Department of Clinical Sciences, Faculty of Veterinary Medicine, Garmsar Branch of Islamic Azad University, Semnan, Iran; ^4^Department of Epidemiology, School of Public Health, Kermanshah University of Medical Sciences, Kermanshah, Iran; ^5^Infectious Diseases Research Center, Health Policy and Promotion Institute, Kermanshah University of Medical Sciences, Kermanshah, Iran

**Keywords:** Gender disparity, Mortality, Socioeconomic characteristics, Substance-related disorders, Substance use, Iran

## Abstract

**Background:**

Illicit drug use is a serious multi-factorial public health challenge globally, especially in the Eastern Mediterranean Region (EMR). In 2019, the disability-adjusted life year rate for drug use disorders in the EMR rose by approximately 40%, whereas the global rate increased by less than 25%. Despite this growing burden, limited data exist on gender patterns of drug-related mortality and its associated socio-economic factors at the national level in Iran. This study evaluated socioeconomic factors contributing to gender disparities in illicit drug-related mortality across provinces of Iran.

**Study Design:**

A national ecological study.

**Methods:**

A secondary analysis of data from the Iranian Forensic Medicine Organization’s national registry was conducted from March 2022 to March 2024. Descriptive statistics were calculated for socioeconomic indicators (happiness, life satisfaction, literacy rate, unemployment, economic participation, and gross domestic product) across provinces of Iran to assess gender disparities in illicit drug-related mortality. Bivariate and multivariate regression analyses were performed to examine the relationship between these indicators and the sex ratio of illicit drug-related deaths.

**Results:**

The sex ratio of illicit drug-related deaths was 7.20, indicating a considerable gender disparity. According to the final model, greater female economic participation (β=-0.517, *P*=0.018) and higher GDP (β=-1.196, *P*=0.002) were significantly correlated with a lower gender gap in illicit drug-related mortality across provinces in Iran.

**Conclusion:**

At the provincial level, there were noticeable correlations between a narrower gender gap in illicit drug-related mortality and both greater female economic participation and higher GDP in Iran.

## Background

 Illicit drug use remains a notable multifactorial public health challenge worldwide. According to evidence, it impacts personal health, community welfare, and economic productivity, particularly in the Eastern Mediterranean Region (EMR).^1,2^ In 2019, the disability-adjusted life year rate for drug use disorders in the EMR had increased by approximately 40% since 1990, compared to a global increase of less than 25%.^3^ The EMR countries encounter distinct obstacles that intensify the drug use crisis (e.g., armed conflicts, political volatility, and economic inequalities).^3,4^ These complex conditions contribute to the widespread production, trafficking routes, marketing, and consumption of illegal drugs in the region.^5^ Among EMR countries, the strategic location of Iran between Afghanistan, the global leader in opium production, and key drug consumer markets heightens its susceptibility to drug-related challenges, including public health crises and social consequences.^4,6^

 However, illicit drug-related mortality data in Iran are often underreported or misclassified.^7^ Based on estimates from 2017, the incidence of drug-related mortality ranged from 3.8^9,10^ to 4.1^10^ per 100,000 people. Prior studies also highlight substantial geographic variations and gender disparities in fatal drug overdoses.^6,7,11^ In Iran, higher drug-related mortality rates have been associated with several risk factors, including lower educational level, history of overdose, suicide attempts, psychiatric hospitalization, incarceration, and family substance abuse.^9,12,13^ While the incidence of fatal overdose is generally lower among women than men,^6,11^ the underlying risk factors considerably differ between genders. Women are influenced by a combination of social determinants, hormonal and biological variations, and psychological influences.^14^ Additionally, mental health disorders, which are more prevalent among women, may contribute to increased drug use and exacerbate women’s vulnerability to drug overdose.^14^ Moreover, women frequently face challenges in accessing treatment for substance use disorders.^4,5,14^

 Since the mid-to-late 1990s, Iran has implemented harm reduction strategies.^15,16^ These initiatives later expanded to include opioid agonist therapies. Despite these efforts, the prevalence of drug use disorders and related mortality continues to rise.^3,10^ Concurrently, the national drug policies of Iran heavily emphasize strict law enforcement, border control, and the criminalization of illicit drug use. Nonetheless, they also incorporate preventive measures, community engagement, and expanded treatment, rehabilitation, and harm reduction services, reflecting a broadly comprehensive approach.^17^ However, it remains unclear how these broad national policies interact with local socioeconomic contexts and adequately address gender-specific vulnerability, particularly among women. Given the above-mentioned discussions, socioeconomic inequality is a critical issue in public health, especially in the context of drug use mortality. Therefore, this study aims to investigate the socioeconomic factors, including happiness, life satisfaction, literacy rate, unemployment, economic participation, and gross domestic product (GDP), contributing to gender disparities in illicit drug-related mortality across provinces of Iran.

## Methods

###  Study Design and Setting

 This cross-sectional ecological study was conducted based on aggregated data at the province level, using information from all 31 provinces of Iran from March 2022 to March 2024. This period was selected because data were only available for a narrow two-year window due to restrictions in data availability. The ecological unit of analysis was the province. The study sought to describe geographic variations in gender differences in drug-related mortality, expressed as the male-to-female mortality ratio (the number of male deaths per one female death), and to examine provincial socioeconomic determinants of these disparities. The ecological nature of our data implies that the observed associations are at the provincial level; thus, inferences about individual-level risk are not warranted to avoid the ecological fallacy.

###  Data Sources and Description

 The study involved a secondary analysis of national illegal drug-related mortality data.^18^ The data were obtained from the Iranian Forensic Medicine Organization’s national registry for the period of March 2022 to March 2024. Under the laws of Iran, all suspicious or undetermined deaths are investigated by the Forensic Medicine Organization (FMO). Forensic physicians determine the cause of death based on the International Classification of Diseases (ICD) principles and record it as a *Persian text phrase* (e.g., “death due to illegal drug use”) in the official certificate. However, the database records this textual description without its corresponding specific ICD code.^6,18^ Although the dataset contains basic demographic information (age, gender, and province), it lacks both specific toxicology data and key socioeconomic and clinical variables (e.g., educational level, marital status, level of urbanization, history of mental health disorders, and socioeconomic status). Provincial covariates serving as indicators of socioeconomic development and well-being were incorporated to address this limitation at the ecological level. For example, (1) GDP per capita was calculated by dividing the real GDP for the year 2021, as obtained from the Statistical Center of Iran, by the provincial population. The values are expressed in million Iranian rials per person to account for differences in population size across provinces. Other covariates^19^ included (2) economic participation rate, defined as the percentage of individuals aged 15 years and older who were either employed or actively looking for work, and (3) the happiness data originated from a large-scale national survey targeting individuals aged 18–65 years across all provinces. Then, a multi-stage random sampling design was used to recruit approximately 24,000 participants. In addition, happiness was measured with the question, “*Are you a lively and cheerful person?*” on a five-point Likert-type scale ranging from *Very Little* (1 point) to *Very Much* (5 points), where higher scores indicated a greater level of happiness within each province. The life-satisfaction data were separately extracted from the 2015 Iran Multiple Indicator Demographic and Health Survey, which provides standardized provincial estimates.

###  Outcome Definition

 The primary outcome was the sex ratio of drug-related mortality at the provincial level, which was calculated as the ratio of the number of male deaths to the number of female deaths within the same province. Higher values demonstrate a greater predominance of male mortality relative to female mortality.

###  Data Quality Assurance

 Before the analysis, all datasets were carefully reviewed by the investigator in order to ensure completeness and validity. It should be noted that no province-level observations were excluded due to missing outcome data.

###  Statistical Analysis

 Descriptive statistics, including means, standard deviations (SD), and ranges, were calculated for the outcome variable and continuous covariates. Pairwise associations between each covariate and the sex ratio were assessed using simple linear regression. A two-step, data-driven strategy was used to build the multivariable model; significant variables (*P*< 0.05) in bivariate analyses, such as real GDP (logarithmic scale), happiness, and female economic participation, were included in the final multivariable weighted linear regression model. To account for population differences, the model was fitted with the sex ratio as the dependent variable and provincial population size as weights (pweight = population). Robust variance estimators (VCE = robust) were utilized to address potential heteroskedasticity. Given the relatively small number of provincial observations (n = 31), bootstrap resampling with 1,000 replications was employed to assess the stability and robustness of the regression coefficients, providing empirical estimates of standard errors and confidence intervals. Moreover, multicollinearity was assessed using variance inflation factors. The results indicated that the variance inflation factor values for all variables were below 2. All statistical analyses were performed using Stata (version 18, Stata Corp, College Station, TX), and a *P*-value < 0.05 was considered statistically significant.

## Results

 The descriptive statistics for the 31 provinces of Iran provided an overview of key socioeconomic and demographic variables relevant to gender disparities in drug-related mortality (Table 1). In 2021, the mean real GDP across provinces was 1,011.72 units, with a high standard deviation (993.7), demonstrating substantial regional economic disparities. In addition, the wide observed range (256.8 to 5,435.3) reflected uneven economic distribution. The average happiness score was 4.69, with considerable variability across provinces (SD = 6.98). Gender-specific labour indicators revealed marked differences, as female economic participation was substantially lower (mean = 13.49%) than male participation (mean = 62.89%). Similarly, women reported lower life satisfaction (mean = 1.23) compared with men (mean = 1.46). Additionally, literacy rates represented persistent educational gaps, with female literacy averaging 79.68% and male literacy 87.54%. Furthermore, unemployment rates reflected further disparities, being notably higher among women (mean = 19.33%) than among men (mean = 9.84%). Based on the results, the gender inequality index averaged near zero (mean = -0.06), although its wide range suggests substantial interprovincial differences. On average, the sex ratio of drug-related deaths was 7.20 (SD = 2.82), indicating a considerable gender gap in mortality.

**Table 1:** Descriptive Statistics of Socioeconomic Indicators Across 31 Provinces of Iran

**Variables**	**Mean**	**SD**	**Min.**	**Max.**
Real GDP	1011.72	993.7	256.8	5435.3
Happiness	4.69	6.98	3.12	42.3
Economic participation (female)	13.49	3.05	8.1	21
Economic participation (male)	62.89	3.55	51.4	69.5
Life satisfaction (female)	1.23	0.07	1.1	1.38
Life satisfaction (male)	1.46	0.1	1.24	1.67
Literacy rate (female)	79.68	4.7	65.4	88.6
Literacy rate (male)	87.54	2.72	77.7	92.3
Unemployment (female)	19.33	5.88	8.9	33.5
Unemployment (male)	9.84	2.58	5.5	16.2
Gender inequality	-0.06	2.11	-4.19	3.72

*Note*. GDP: Gross domestic product; SD: Standard deviation; Min.: Minimum; Max.: Maximum.

 The bivariate regression analysis exploring the association between socioeconomic indicators and the sex ratio of drug-related mortality across 31 provinces of Iran yielded several key findings(Table 2). Real GDP (logarithmic scale) demonstrated a significant negative association (β = -0.918, *P*= 0.020), explaining approximately 9.2% of the variance. Likewise, happiness showed a significant negative association (β = -0.075, *P*< 0.001), accounting for 3.5% of the variance. Moreover, female economic participation exhibited a significant negative association (β = -0.440, *P*= 0.017), implying 23.5% of the variance, while male economic participation was not significant (β = -0.058, *P*= 0.641). Neither female nor male life satisfaction scores represented significant associations. Similarly, female literacy (β = -0.004, *P*= 0.963) and male literacy (β = 0.080, *P*= 0.580) were non-significant predictors. Further, female unemployment (β = 0.052, *P*= 0.429), male unemployment (β = -0.171, *P*= 0.434), and the gender inequality index (β = 0.176, *P*= 0.430) all failed to reach statistical significance. None of the remaining variables demonstrated statistically significant associations with the sex ratio of drug-related mortality.

**Table 2:** Bivariate Regression Results for Predictors of the Sex Ratio in Illicit Drug-Related Mortality

**Variables**	**Beta Coefficient**	**SE**	***P*** ** Value**	**R^2^**
Real GDP (logarithmic scale)	-0.918	0.374	0.020	0.091
Happiness	-0.075	0.014	0.001	0.035
Economic participation (female)	-0.440	0.174	0.017	0.235
Economic participation (male)	-0.058	0.124	0.641	0.006
Life satisfaction (female)	2.760	6.021	0.649	0.004
Life satisfaction (male)	1.455	4.863	0.767	0.003
Literacy rate (female)	-0.004	0.092	0.963	0.000
Literacy rate (male)	0.080	0.143	0.580	0.006
Unemployment (female)	0.052	0.065	0.429	0.012
Unemployment (male)	-0.171	0.215	0.434	0.025
Gender inequality	0.176	0.220	0.430	0.017

*Note*. GDP: Gross domestic product; SE: Standard error.

 Logarithmic GDP, female economic participation, and happiness (as three key variables) were included in the model in a multivariate regression analysis aimed at identifying socioeconomic determinants of the sex ratio in drug-related mortality across 31 provinces of Iran (Table 3). The final model collectively explained 41.8% of the variance in the sex ratio of drug-related mortality, indicating a substantial overall explanatory contribution. Real GDP (logarithmic scale) was the strongest predictor and showed a meaningful negative correlation with the sex ratio (β = −1.196, *P*= 0.002). This finding confirms that provinces with higher economic output tend to exhibit a narrower gender gap in drug-related mortality, meaning that the disparity between male and female mortality rates is smaller in economically stronger provinces. Similarly, female economic participation was a significant negative predictor of the sex ratio (β = −0.517, *P*= 0.018). This suggests that greater integration of women into the provincial economy is correlated with a reduced gender gap in drug-related mortality, reflecting a less pronounced male excess in mortality. In contrast, the correlation between provincial happiness and the outcome was non-significant (β = 0.011, *P*= 0.995), representing no meaningful relationship with the sex ratio. Overall, the analysis highlights that macroeconomic conditions captured by provincial GDP and female economic participation are strongly correlated with narrower gender gaps in drug-related mortality at the population level. In this study, although bootstrap resampling was used to improve the reliability of coefficient estimates, the relatively small number of provinces still limits statistical power. Accordingly, the results should be interpreted with caution.

**Table 3:** Multivariate Regression Model Predicting the Sex Ratio in Illicit Drug-Related Mortality

**Variables**	**Beta Coefficient**	**SE**	***P*** ** Value**	**R^2^**
Real GDP (logarithmic scale)	-1.196	0.379	0.002	0.418
Happiness	0.011	1.575	0.995	
Economic participation (female)	-0.517	0.218	0.018	

*Note*. GDP: Gross domestic product; SE: Standard error.

**Figure 1:**
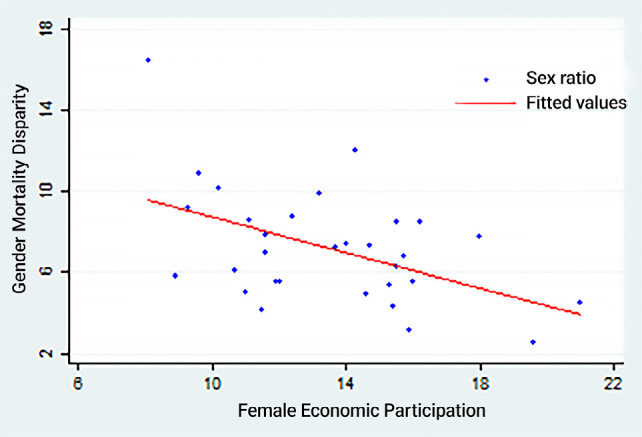
The Ecological Association Between Female Economic Participation and the Gender Disparity in Illicit Drug-Related Mortality Across Different Provinces of Iran

Figure 1 illustrates the ecological association between female economic participation and the gender disparity in illicit drug-related mortality across provinces of Iran. The negative slope of the regression line indicates a statistically significant inverse association (slope = -0.48, *P*= 0.005), suggesting that higher provincial female labor force participation was significantly associated with a narrower gender disparity in mortality.

## Discussion

 Data on drug-related mortality in the EMR are limited; however, Iran provides more comprehensive data on drug use disorders compared to other regional countries.^20-22^ These robust data highlight the significant role of mental health and drug use disorders in the burden of disease in Iran.^18,23^ Although drug-related mortality in Iran has been examined from various perspectives, its analysis in relation to macroeconomic and social indicators has received limited attention.

 Our analysis of socio-economic factors across Iran’s provinces revealed significant gender disparities in illicit drug-related mortality, with a mean sex ratio of 7.20, during the study period. Key findings indicated that greater female economic participation (β = -0.440, *P*= 0.017) and higher real GDP (β = -0.918, *P*= 0.020) were both inversely correlated with narrower gender gaps in drug-related deaths. The observed inverse association may be partly explained by women’s unique risk factors for overdose, including social determinants, hormonal and biological variations, and psychological influences.^14^ It should be noted that mental health disorders, which are more prevalent among women, may exacerbate vulnerability to drug use and overdose.^14,24^ In this context, the strong, significant relationship observed between drug abuse and suicidal ideation further underscores how psychological vulnerabilities amplify overdose risks, particularly among women.^25^ Furthermore, women often face challenges in accessing treatment for drug and substance use disorders due to socioeconomic and systemic barriers, which are compounded by cultural stigma and gender-specific barriers that diminish the effectiveness of harm reduction programs.^4,5,14,15^ These factors emphasize the need for targeted interventions to address women’s specific vulnerabilities and improve access to care. In addition, several hypothetical mechanisms may explain the narrower gender gap observed with higher female economic participation and stronger provincial GDP. First, greater financial independence and mobility may increase women’s exposure to mixed-gender social or occupational environments where substances are more accessible. Second, stronger economic conditions may facilitate a shift from traditional to more modern behavioural norms, increasing openness to risk-taking behaviours historically more common among men. Third, in more urbanized and economically advantaged settings, reduced social and familial control may lower external constraints on experimenting with substances. Nonetheless, these explanations remain speculative and hypothesis-generating. Therefore, future individual-level studies are needed to examine their validity.

 Additionally, happiness showed a strong inverse association with the sex ratio (β = -0.075, *P*< 0.001) in bivariate analysis, though its effect diminished in the multivariate model, possibly due to increased variance. Our analysis indicated that female economic participation and real GDP act as qualitative confounders in the relationship between happiness and the sex ratio in drug-related mortality across provinces of Iran. In bivariate regression (Table 2), happiness had a significant negative association with the sex ratio (β = -0.075, *P*< 0.001), demonstrating that higher happiness correlates with a smaller gender gap in drug-related mortality. However, in multivariate regression (Table 3), when female economic participation and real GDP (logarithmic scale) are included, the association shifted to a non-significant positive one (β = + 0.011, *P*= 0.995). This reversal and loss of significance suggest that female economic participation and log-transformed GDP are qualitative confounders, altering the direction and significance of the happiness-sex ratio relationship. The increased standard error in the multivariate model (1.57 vs. 0.014) represents that these confounders account for additional variance, potentially masking happiness’s direct effect. Briefly, the shift in direction and loss of significance when real GDP and female economic participation were included suggest that these socioeconomic indicators act as qualitative confounders, thereby masking the direct impact of happiness. Furthermore, national evidence from the Iranian adult population revealed a considerable association between happiness and several socioeconomic factors, including age, education, occupation, and income.^26^ Gender-stratified analysis demonstrated that age, education, occupation, and income remained significant predictors of happiness for men. In contrast, for women, only education and income showed noticeable associations with happiness. Additionally, income was identified as a direct predictor of happiness in the studied population.^26^ These findings support the plausibility of confounding, as provinces with higher GDP and female economic participation likely have greater aggregate happiness. It can be cautiously speculated that potential pathways for this confounding effect are through improved economic conditions and associated well-being influencing gender-specific risk behaviors and social dynamics, which can subsequently impact the sex ratio in illicit drug-related mortality.

 Socioeconomic disparities are evident in drug and substance use. According to the Iran Mental Health Survey, the prevalence of drug use disorders is more pronounced among lower socioeconomic groups. This aligns with the findings of a study on male medical students in Iran, which identified maternal educational level, living place, economic status, and parents’ divorce as the most influential predictive factors for drug abuse.^27^ Improving the socioeconomic status of households, particularly for men, divorced or widowed individuals, and the unemployed, may help mitigate this disparity.^28^ Consistent with this evidence, a comprehensive time-series analysis indicated that drug-related mortality in Iran is significantly influenced by key socioeconomic factors, such as GDP and literacy rate, both of which display strong associations with drug-related mortality.^19^ These findings, reported without gender distinction, highlight the long-term impact of macroeconomic and social indicators on drug-related mortality.^19^ Conversely, individuals in the lowest income group were more likely to report substance abuse issues compared to those in the highest income group.^29,30^ Similarly, socioeconomic factors, especially financial status, are strongly linked to treatment dropout rates among those with drug and substance use disorders.^31^

 Despite the comprehensive harm reduction strategies of Iran, challenges (e.g., the increasing use of new synthetic substances and persistent socioeconomic barriers) continue to hinder progress in reducing addiction rates and drug-related mortality.^4,5,14,15^ These factors may partially explain the gender disparities observed in our study and underscore the need for adaptive policies addressing emerging drug trends and socioeconomic inequities. Thus, future studies should rigorously examine the interplay of socioeconomic determinants, cultural stigma, and access to mental health services in driving gender-specific outcomes while evaluating the effectiveness of tailored interventions in order to reduce drug-related mortality.

 The current study had several limitations. The analysis was potentially limited by the temporal misalignment of certain covariates (e.g., labor force participation and happiness index), which were sourced from earlier years than the outcome data. While these were the most recent reliable provincial-level data available, this discrepancy introduces the potential for temporal confounding if the relative standing of provinces on these measures has changed over time.Furthermore,the dataset lacked information on mortality associated with alcohol consumption. Additionally, due to the constraints of the available data, individual-level sociodemographic variables (e.g., marital status, occupation, and educational attainment) were not included, which could have provided insights into correlations with gender-specific drug-related mortality. Moreover, in rural and remote regions, some deaths may not have been reported to the FMO for further examination, potentially due to limited access to forensic services and heightened stigma surrounding drug use, leading to possible underreporting. In addition, the dataset lacked detailed information regarding the manner of death (e.g., whether it resulted from accidental or intentional poisoning) or indirect consequences of illicit drug use (e.g., road traffic injuries). Finally, due to the restricted access to toxicology data from the FMO, the researchers were unable to analyze gender-specific differential mortality by specific drug types.

**Highlights**

- The male-to-female ratio of drug-related deaths was 7.20, highlighting a substantial gender disparity in mortality across Iranian provinces.
- Higher female economic participation was significantly correlated with a narrower gender gap in illicit drug-related deaths (β = -0.517, *P*= 0.018).
- Higher provincial real GDP was noticeably correlated with a narrower gender gap in illicit drug-related mortality (β = -1.196, *P*= 0.002).
- Several hypothetical mechanisms could explain the narrower gender gap observed with higher female economic participation and stronger provincial GDP.

## Conclusion

 The findings of this ecological study revealed that greater female economic participation and higher real GDP at the provincial level were significantly correlated with a narrower gender gap in illicit drug-related mortality in Iran. While causality cannot be inferred from these findings, they suggest that public health strategies should prioritize women’s economic empowerment and strengthen economic infrastructure. There is a critical need to implement evidence-based, gender-responsive harm reduction interventions in order to address these disparities effectively. Eventually, a deeper understanding of the underlying mechanisms is essential to guide sustainable policy and public health strategies.

## Artificial Intelligence Use Statement

 The authors declare that artificial intelligence tools were used solely for language editing and grammatical improvement of the manuscript. The authors exclusively analyzed the data, interpreted the results, drew conclusions, and generated original scientific content. They take full responsibility for the integrity, accuracy, and originality of the work.

## Competing Interests

 Dr. Seyed Amirhosein Mahdavi is one of the vice-chancellors of the Iranian Legal Medicine Organization. Other authors declare they have no conflict of interests.

## Ethical Approval

 This study was based on a secondary analysis of national illegal drug-related mortality data. The study protocol of the original data collection was reviewed and approved by the Ethics Committee of the Iranian Legal Medicine Organization (reference No. IR.LMO.REC.1403.004). Considering that the dataset was fully anonymized and de-identified prior to analysis, no additional ethical approval or informed consent was required for the present study.

## Funding

 This paper received no specific grant from any funding agency in public, commercial, or not-for-profit sectors.
